# Crystal structure of [3-amino-2-(phenyl­diazenyl)­pyridine]chlorido­(η^6^-*p*-cymene)­ruthenium(II) chloride

**DOI:** 10.1107/S2056989015017466

**Published:** 2015-09-26

**Authors:** Kanidtha Hansongnern, Supojjanee Sansook, Thassani Romin, Arunpatcha Nimthong Roldan, Chaveng Pakawatchai

**Affiliations:** a Department of Chemistry, Faculty of Science, Prince of Songkla University, Hat Yai, Songkhla 90112, Thailand; b Department of Chemistry, Youngstown State University, 1 University Plaza, 44555, Youngstown, OH, USA

**Keywords:** crystal structure, 3-amino-2-(phenyl­azo)pyridine, ruthenium complex, N—H⋯Cl hydrogen bonds

## Abstract

The title compound, [RuCl(C_10_H_14_)(C_11_H_10_N_4_)]Cl is an Ru^II^ complex in which an η^*6*^-*p*-cymene ligand, two N atoms of 3-amino-2-(phenyl­azo)pyridine and one Cl ion form a piano-stool coordination environment around the metal ion. In the crystal structure, N—H⋯Cl hydrogen bonds play an important role in the formation of the supramolecular zigzag chain along the *a*-axis direction. Disorder is observed for the isopropyl group with site-occupancy factors refined to 0.78 (5) and 0.22 (5).

## Related literature   

For anti­cancer activity of organometallic ruthenium complexes, see: Almodares *et al.* (2014 ▸); Stepanenko *et al.* (2011 ▸). For the use of a similar azo­pyridine ligand to stabilize ruthenium complexes, see: Velders *et al.* (2000 ▸). For related η^*6*^-*p*-cymene ruthenium complexes, see: Singh *et al.* (2002 ▸), Kumar *et al.* (2008 ▸). For the crystal structure of an η^*6*^-*p*-cymene ruthenium complex with an azo­pyridine ligand, see: Dougan *et al.* (2006 ▸).
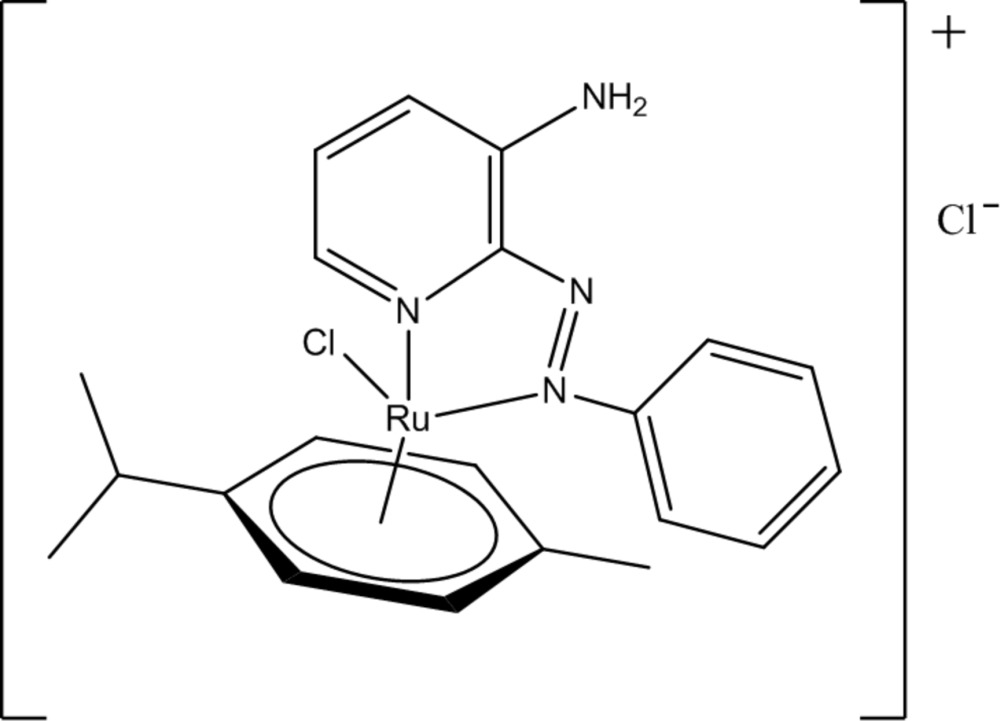


## Experimental   

### Crystal data  

[RuCl(C_10_H_14_)(C_11_H_10_N_4_)]Cl
*M* *_r_* = 504.41
Orthorhombic, 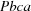

*a* = 8.9642 (8) Å
*b* = 17.6283 (16) Å
*c* = 26.976 (3) Å
*V* = 4262.8 (7) Å^3^
*Z* = 8
Mo *K*α radiation
μ = 1.00 mm^−1^
*T* = 293 K
0.44 × 0.10 × 0.07 mm

### Data collection  

Bruker SMART APEX CCD Diffractometer
Absorption correction: multi-scan (*SADABS*; Bruker, 2003 ▸) *T*_min_ = 0.693, *T*_max_ = 1.000
40186 measured reflections
5127 independent reflections
4242 reflections with *I* > 2σ(*I*)
*R*_int_ = 0.069

### Refinement  

*R*[*F* ^2^ > 2σ(*F* ^2^)] = 0.074
*wR*(*F* ^2^) = 0.136
*S* = 1.12
5127 reflections
265 parameters
15 restraints
H-atom parameters constrained
Δρ_max_ = 0.71 e Å^−3^
Δρ_min_ = −1.61 e Å^−3^

### 

Data collection: *SMART* (Bruker, 1998 ▸); cell refinement: *SAINT* (Bruker, 2003 ▸); data reduction: *SAINT*; program(s) used to solve structure: *SHELXS97* (Sheldrick, 2008 ▸); program(s) used to refine structure: *SHELXL2012* (Sheldrick, 2015 ▸) and *SHELXLE* (Hübschle *et al.*, 2011 ▸); molecular graphics: *Mercury* (Macrae *et al.*, 2008 ▸); software used to prepare material for publication: *SHELXL97* (Sheldrick, 2008 ▸) and *publCIF* (Westrip, 2010 ▸).

## Supplementary Material

Crystal structure: contains datablock(s) I. DOI: 10.1107/S2056989015017466/zq2235sup1.cif

Structure factors: contains datablock(s) I. DOI: 10.1107/S2056989015017466/zq2235Isup2.hkl

**Fig. 1.**
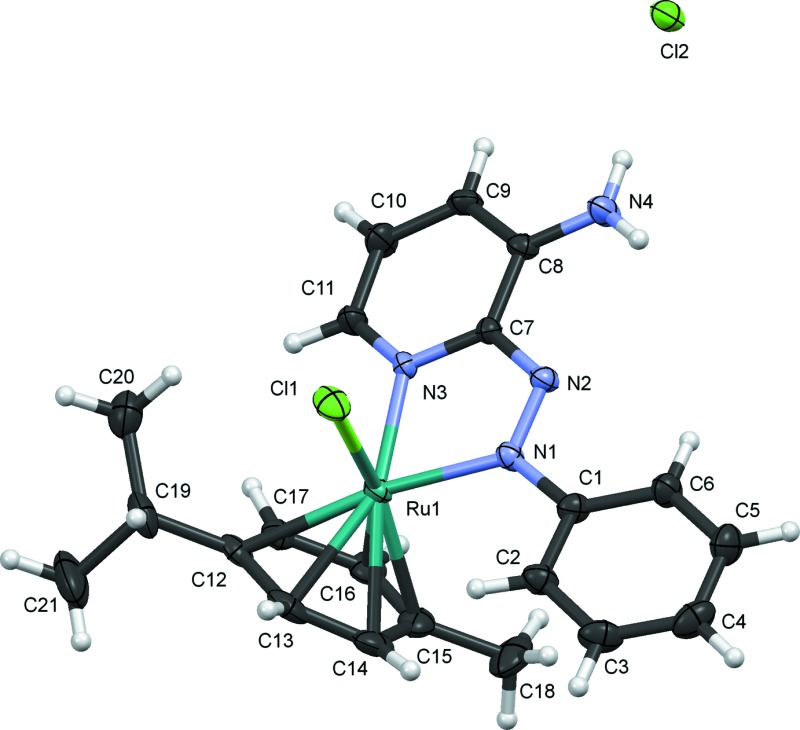
The molecular structure of the title compound showing the atom-labelling scheme. Displacement ellipsoids are drawn at the 30% probability level.

**Fig. 2.**
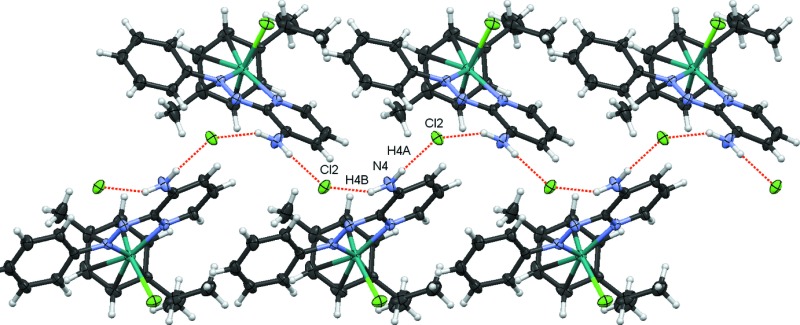
Crystal packing of the title compound showing the N—H···Cl hydrogen bonds along the a axis.

. DOI: 10.1107/S2056989015017466/zq2235fig1.tif
The mol­ecular structure of the title compound showing the atom-labelling scheme. Displacement ellipsoids are drawn at the 30% probability level.

a . DOI: 10.1107/S2056989015017466/zq2235fig2.tif
Crystal packing of the title compound showing the N—H⋯Cl hydrogen bonds along the *a* axis.

CCDC reference: 1425731

Additional supporting information:  crystallographic information; 3D view; checkCIF report

## Figures and Tables

**Table 1:** Hydrogen-bond geometry (, )

*D*H*A*	*D*H	H*A*	*D* *A*	*D*H*A*
N4H4*A*Cl2	0.86	2.34	3.200(5)	176
N4H4*B*Cl2^i^	0.86	2.37	3.152(5)	151

Symmetry code: (i) 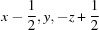.

## supplementary crystallographic information

### S0.1. Synthesis and crystallization

3-amino-2-(phenyl­azo)pyridine (*3aazpy*) was prepared by condensation of 2,3-di­amino-pyridine (14 mg, 2 mmol) with nitroso­benzene (217 mg, 2.1 mmol) in a mixture of 30 M NaOH (3 mL) and 35 mL of benzene solution. The reaction mixture was stirred and heated under reflux for 14 h. The product was extracted many times with benzene to obtain the brown solution. Then the volume was reduced to 3 mL. The residue was purified by column chromatography. The red-orange band was collected and evaporated to dryness (yield : 37%).

The title compound was obtained by the following procedure: [(η*^6^*-*p*-cym)RuCl_2_]_2_ (0.05 mmol) was added to a THF solution of *3aazpy* (0.1 mmol); the solution color change from red to purple. The solution was stirred at ambient temperature for 2 h. The precipitate was collected by filtration, washed with a small amount of THF. Monocrystals were obtained by diffusion of ether into a di­chloro­methane solution of the complex (yield: 82%).

### S0.2. Refinement

H atoms bonded to C and N atoms were included in calculated positions and were refined with a riding model using distances of 0.93 Å (aryl H), and *U*_iso_(H) = 1.2*U*_eq_(C); 0.98 Å (CH) and *U*_iso_(H) = 1.5*U*_eq_(C); 0.96 Å (CH_3_) and *U*_iso_(H) = 1.5*U*_eq_(C); 0.86 Å (NH_2_), and *U*_iso_(H) = 1.2*U*_eq_(N). A disorder is observed for the iso­propyl group with site-occupancy factors refined to 0.78 (5) and 0.22 (5).

### S1. Results and discussion

Organometallic ruthenium complexes have gained much inter­est due to promising anti­cancer activity (Almodares *et al.*, 2014; Stepanenko *et al.*, 2011). These arene complexes consist of a chelating ligand and one chloride ion. In this work, a new ruthenium(II) complex of this type is reported.

The title complex exists as a half sandwich complex with the neutral arene ring bonded to the ruthenium center along with two N atoms from 3-amino-2-(phenyl­azo)pyridine (*3aazpy*) and one chloride ion (Fig. 1). The *3aazpy* ligand contains the –N=N—C=N linkage which is similar to 2-(phenyl­azo)pyridine (*azpy*). Azo ligands of this type can stabilize metals in their lower oxidation states (Velders *et al.*, 2000). The ruthenium atom is π-bonded to the *p*-cymene ligand with an average Ru—C bond length of 2.215 (6) Å (range 2.185 (6) - 2.243 (5) Å) similar to those observed in related η*^6^*-*p*-cymene ruthenium complexes (Singh *et al.*, 2002, Kumar *et al.*, 2008). The *p*-cymene ring is almost planar and the C—C bond lengths within the ring are in the range 1.376 – 1.435 Å. The ruthenium center is also coordinated to N1 (azo moiety, 2.078 (4) Å) and to N3 (pyridine, 2.077 (4) Å) of *3aazpy*. These Ru—N bond lengths are longer than those in [(η*^6^*-*p*-cymene)Ru(*azpy*)Cl](PF_6_) (Dougan *et al.*, 2006). It indicates that *azpy* is a better ligand to stabilize the ruthenium(II) center than *3aazpy*. Meanwhile, the bite angle of 75.6 (2)° of the chelate ligand and the Ru—Cl bond distance of 2.3938 (15) Å are comparable to those in [(η*^6^*-*p*-cym)Ru(*azpy*)Cl](PF_6_). In the crystal structure, chloride ions are linked with the complex molecule through N4—H4*A*···Cl2^i^ and N4—H4*B*···Cl2 hydrogen bonds leading to the formation of a 1-D zigzag chain along the *a*-axis (see Table 1 and Fig. 2). A disorder is observed for the iso­propyl group with site-occupancy factors refined to 0.78 (5) and 0.22 (5).

### Crystal data

**Table:**

[RuCl(C_10_H_14_)(C_11_H_10_N_4_)]Cl	*D*_x_ = 1.572 Mg m^−^^3^
*M**_r_* = 504.41	Mo *K*α radiation, λ = 0.71073 Å
Orthorhombic, *P**b**c**a*	Cell parameters from 7302 reflections
*a* = 8.9642 (8) Å	θ = 2.3–27.9°
*b* = 17.6283 (16) Å	µ = 1.00 mm^−^^1^
*c* = 26.976 (3) Å	*T* = 293 K
*V* = 4262.8 (7) Å^3^	Block, colorless
*Z* = 8	0.44 × 0.10 × 0.07 mm
*F*(000) = 2048	

### Data collection

**Table:**

Bruker SMART APEX CCD Diffractometer	4242 reflections with *I* > 2σ(*I*)
Radiation source: sealed tube	*R*_int_ = 0.069
φ and ω scans	θ_max_ = 28.0°, θ_min_ = 1.5°
Absorption correction: multi-scan (*SADABS*; Bruker, 2003)	*h* = −11→11
*T*_min_ = 0.693, *T*_max_ = 1.000	*k* = −23→23
40186 measured reflections	*l* = −35→35
5127 independent reflections	

### Refinement

**Table:**

Refinement on *F*^2^	Primary atom site location: structure-invariant direct methods
Least-squares matrix: full	Secondary atom site location: difference Fourier map
*R*[*F*^2^ > 2σ(*F*^2^)] = 0.074	Hydrogen site location: inferred from neighbouring sites
*wR*(*F*^2^) = 0.136	H-atom parameters constrained
*S* = 1.12	*w* = 1/[σ^2^(*F*_o_^2^) + (0.0276*P*)^2^ + 25.095*P*] where *P* = (*F*_o_^2^ + 2*F*_c_^2^)/3
5127 reflections	(Δ/σ)_max_ = 0.002
265 parameters	Δρ_max_ = 0.71 e Å^−^^3^
15 restraints	Δρ_min_ = −1.61 e Å^−^^3^

### Special details

**Table:**

Geometry. All e.s.d.'s (except the e.s.d. in the dihedral angle between two l.s. planes) are estimated using the full covariance matrix. The cell e.s.d.'s are taken into account individually in the estimation of e.s.d.'s in distances, angles and torsion angles; correlations between e.s.d.'s in cell parameters are only used when they are defined by crystal symmetry. An approximate (isotropic) treatment of cell e.s.d.'s is used for estimating e.s.d.'s involving l.s. planes.

### Fractional atomic coordinates and isotropic or equivalent isotropic displacement parameters (Å^2^)

**Table:**

	*x*	*y*	*z*	*U*_iso_*/*U*_eq_	Occ. (<1)
Ru1	0.62601 (5)	0.03668 (2)	0.38760 (2)	0.03006 (13)	
Cl1	0.73313 (19)	−0.03312 (8)	0.45488 (5)	0.0473 (4)	
Cl2	0.98928 (17)	−0.30554 (8)	0.21404 (6)	0.0434 (4)	
N1	0.5259 (5)	−0.0663 (2)	0.37034 (16)	0.0298 (9)	
N2	0.5993 (5)	−0.1133 (2)	0.34347 (16)	0.0310 (10)	
N3	0.7791 (5)	−0.0169 (2)	0.34159 (16)	0.0283 (9)	
N4	0.7774 (5)	−0.2032 (3)	0.28221 (19)	0.0450 (13)	
H4A	0.8322	−0.2296	0.2625	0.054*	
H4B	0.6955	−0.2218	0.2935	0.054*	
C1	0.3803 (6)	−0.0944 (3)	0.3838 (2)	0.0352 (12)	
C2	0.3052 (7)	−0.0609 (3)	0.4228 (2)	0.0410 (14)	
H2	0.3527	−0.0249	0.4423	0.049*	
C3	0.1594 (7)	−0.0812 (4)	0.4325 (3)	0.0511 (17)	
H3	0.1065	−0.0564	0.4574	0.061*	
C4	0.0923 (8)	−0.1376 (4)	0.4057 (3)	0.0571 (19)	
H4	−0.0057	−0.1514	0.4125	0.069*	
C5	0.1712 (8)	−0.1743 (4)	0.3684 (3)	0.0542 (18)	
H5	0.1273	−0.2140	0.3510	0.065*	
C6	0.3139 (7)	−0.1520 (3)	0.3573 (2)	0.0413 (14)	
H6	0.3658	−0.1757	0.3318	0.050*	
C7	0.7341 (6)	−0.0871 (3)	0.32750 (18)	0.0270 (10)	
C8	0.8194 (6)	−0.1338 (3)	0.2952 (2)	0.0311 (11)	
C9	0.9527 (6)	−0.1018 (3)	0.2774 (2)	0.0389 (13)	
H9	1.0131	−0.1291	0.2558	0.047*	
C10	0.9932 (7)	−0.0306 (3)	0.2921 (2)	0.0477 (15)	
H10	1.0801	−0.0090	0.2796	0.057*	
C11	0.9061 (6)	0.0096 (3)	0.3252 (2)	0.0396 (14)	
H11	0.9388	0.0568	0.3361	0.048*	
C12	0.6938 (7)	0.1496 (3)	0.4186 (2)	0.0374 (13)	
C13	0.5524 (8)	0.1272 (3)	0.4388 (3)	0.0495 (17)	
H13	0.5459	0.1158	0.4743	0.059*	
C14	0.4329 (8)	0.1095 (3)	0.4089 (3)	0.0565 (19)	
H14	0.3456	0.0853	0.4240	0.068*	
C15	0.4465 (8)	0.1082 (3)	0.3565 (3)	0.0549 (18)	
C16	0.5835 (7)	0.1301 (3)	0.3363 (2)	0.0447 (15)	
H16	0.6012	0.1223	0.3008	0.054*	
C17	0.7038 (7)	0.1516 (3)	0.3669 (2)	0.0382 (13)	
H17	0.8028	0.1568	0.3518	0.046*	
C18	0.3198 (8)	0.0843 (5)	0.3244 (3)	0.081 (3)	
H18A	0.3575	0.0676	0.2929	0.121*	
H18B	0.2536	0.1264	0.3195	0.121*	
H18C	0.2667	0.0436	0.3401	0.121*	
C19	0.8212 (9)	0.1721 (4)	0.4513 (3)	0.0580 (19)	0.78 (5)
H19	0.7999	0.1522	0.4845	0.070*	0.78 (5)
C20	0.9735 (12)	0.1408 (13)	0.4358 (7)	0.066 (4)	0.78 (5)
H20A	0.9682	0.0865	0.4335	0.099*	0.78 (5)
H20B	1.0471	0.1547	0.4600	0.099*	0.78 (5)
H20C	1.0006	0.1614	0.4041	0.099*	0.78 (5)
C21	0.824 (3)	0.2586 (7)	0.4555 (12)	0.111 (8)	0.78 (5)
H21A	0.9016	0.2735	0.4781	0.167*	0.78 (5)
H21B	0.7298	0.2763	0.4675	0.167*	0.78 (5)
H21C	0.8437	0.2802	0.4234	0.167*	0.78 (5)
C19B	0.8212 (9)	0.1721 (4)	0.4513 (3)	0.0580 (19)	0.22 (5)
H19B	0.8245	0.1339	0.4777	0.070*	0.22 (5)
C20B	0.974 (4)	0.169 (4)	0.426 (2)	0.066 (4)	0.22 (5)
H20D	0.9845	0.1215	0.4094	0.099*	0.22 (5)
H20E	1.0504	0.1738	0.4510	0.099*	0.22 (5)
H20F	0.9819	0.2098	0.4030	0.099*	0.22 (5)
C21B	0.791 (9)	0.247 (3)	0.477 (3)	0.111 (8)	0.22 (5)
H21D	0.8799	0.2637	0.4939	0.167*	0.22 (5)
H21E	0.7125	0.2405	0.5011	0.167*	0.22 (5)
H21F	0.7618	0.2844	0.4533	0.167*	0.22 (5)

### Atomic displacement parameters (Å^2^)

**Table:**

	*U*^11^	*U*^22^	*U*^33^	*U*^12^	*U*^13^	*U*^23^
Ru1	0.0334 (2)	0.02129 (19)	0.0355 (2)	0.00223 (18)	0.00250 (19)	−0.00160 (17)
Cl1	0.0641 (10)	0.0363 (7)	0.0414 (8)	0.0089 (7)	−0.0093 (7)	0.0017 (6)
Cl2	0.0424 (8)	0.0322 (7)	0.0558 (9)	−0.0014 (6)	0.0150 (7)	−0.0051 (6)
N1	0.033 (2)	0.024 (2)	0.032 (2)	−0.0030 (19)	0.0057 (19)	−0.0007 (17)
N2	0.030 (2)	0.023 (2)	0.040 (2)	0.0006 (18)	0.0072 (19)	−0.0019 (18)
N3	0.028 (2)	0.021 (2)	0.036 (2)	−0.0013 (17)	−0.0003 (19)	−0.0040 (17)
N4	0.039 (3)	0.031 (3)	0.065 (3)	−0.004 (2)	0.019 (3)	−0.011 (2)
C1	0.032 (3)	0.032 (3)	0.041 (3)	−0.002 (2)	0.009 (3)	0.006 (2)
C2	0.043 (3)	0.036 (3)	0.044 (3)	0.002 (3)	0.011 (3)	0.001 (3)
C3	0.049 (4)	0.050 (4)	0.054 (4)	0.006 (3)	0.023 (3)	0.009 (3)
C4	0.036 (4)	0.069 (5)	0.066 (5)	−0.005 (3)	0.006 (3)	0.020 (4)
C5	0.047 (4)	0.047 (4)	0.069 (5)	−0.016 (3)	0.002 (3)	0.001 (3)
C6	0.038 (3)	0.037 (3)	0.049 (4)	−0.008 (3)	0.009 (3)	−0.004 (3)
C7	0.025 (2)	0.024 (2)	0.032 (3)	−0.001 (2)	0.000 (2)	0.000 (2)
C8	0.034 (3)	0.025 (3)	0.035 (3)	0.004 (2)	0.004 (2)	0.002 (2)
C9	0.031 (3)	0.040 (3)	0.046 (3)	0.008 (2)	0.010 (3)	−0.004 (3)
C10	0.036 (3)	0.045 (3)	0.062 (4)	−0.011 (3)	0.014 (3)	−0.006 (3)
C11	0.032 (3)	0.034 (3)	0.053 (4)	−0.004 (2)	0.010 (3)	−0.011 (3)
C12	0.060 (4)	0.015 (2)	0.037 (3)	0.001 (2)	0.004 (3)	0.002 (2)
C13	0.076 (5)	0.021 (3)	0.052 (4)	0.006 (3)	0.020 (4)	−0.004 (3)
C14	0.050 (4)	0.026 (3)	0.093 (6)	0.011 (3)	0.019 (4)	−0.005 (3)
C15	0.048 (4)	0.030 (3)	0.087 (5)	0.011 (3)	−0.014 (4)	0.002 (3)
C16	0.055 (4)	0.031 (3)	0.049 (4)	0.010 (3)	−0.007 (3)	0.008 (3)
C17	0.045 (3)	0.024 (3)	0.045 (3)	0.003 (3)	0.002 (3)	0.006 (2)
C18	0.053 (5)	0.064 (5)	0.126 (8)	0.007 (4)	−0.038 (5)	0.020 (5)
C19	0.089 (6)	0.036 (3)	0.049 (4)	−0.013 (4)	−0.013 (4)	−0.012 (3)
C20	0.066 (6)	0.064 (10)	0.068 (8)	−0.018 (6)	−0.016 (5)	−0.002 (7)
C21	0.147 (15)	0.048 (6)	0.14 (2)	−0.010 (8)	−0.054 (14)	−0.032 (9)
C19B	0.089 (6)	0.036 (3)	0.049 (4)	−0.013 (4)	−0.013 (4)	−0.012 (3)
C20B	0.066 (6)	0.064 (10)	0.068 (8)	−0.018 (6)	−0.016 (5)	−0.002 (7)
C21B	0.147 (15)	0.048 (6)	0.14 (2)	−0.010 (8)	−0.054 (14)	−0.032 (9)

### Geometric parameters (Å, º)

**Table:**

Ru1—N3	2.077 (4)	C10—H10	0.9300
Ru1—N1	2.078 (4)	C11—H11	0.9300
Ru1—C16	2.185 (6)	C12—C17	1.398 (8)
Ru1—C15	2.210 (6)	C12—C13	1.435 (9)
Ru1—C13	2.211 (6)	C12—C19	1.498 (9)
Ru1—C17	2.215 (5)	C13—C14	1.376 (10)
Ru1—C14	2.231 (6)	C13—H13	0.9800
Ru1—C12	2.243 (5)	C14—C15	1.419 (10)
Ru1—Cl1	2.3938 (15)	C14—H14	0.9800
N1—N2	1.283 (6)	C15—C16	1.398 (9)
N1—C1	1.442 (7)	C15—C18	1.489 (10)
N2—C7	1.363 (6)	C16—C17	1.411 (8)
N3—C11	1.307 (7)	C16—H16	0.9800
N3—C7	1.356 (6)	C17—H17	0.9800
N4—C8	1.327 (7)	C18—H18A	0.9600
N4—H4A	0.8600	C18—H18B	0.9600
N4—H4B	0.8600	C18—H18C	0.9600
C1—C6	1.376 (8)	C19—C21	1.529 (12)
C1—C2	1.381 (8)	C19—C20	1.531 (12)
C2—C3	1.381 (8)	C19—H19	0.9800
C2—H2	0.9300	C20—H20A	0.9600
C3—C4	1.368 (10)	C20—H20B	0.9600
C3—H3	0.9300	C20—H20C	0.9600
C4—C5	1.389 (10)	C21—H21A	0.9600
C4—H4	0.9300	C21—H21B	0.9600
C5—C6	1.371 (8)	C21—H21C	0.9600
C5—H5	0.9300	C20B—H20D	0.9600
C6—H6	0.9300	C20B—H20E	0.9600
C7—C8	1.422 (7)	C20B—H20F	0.9600
C8—C9	1.406 (8)	C21B—H21D	0.9600
C9—C10	1.365 (8)	C21B—H21E	0.9600
C9—H9	0.9300	C21B—H21F	0.9600
C10—C11	1.382 (8)		
			
N3—Ru1—N1	75.80 (16)	C9—C10—C11	120.6 (5)
N3—Ru1—C16	94.5 (2)	C9—C10—H10	119.7
N1—Ru1—C16	116.2 (2)	C11—C10—H10	119.7
N3—Ru1—C15	120.9 (2)	N3—C11—C10	121.8 (5)
N1—Ru1—C15	95.7 (2)	N3—C11—H11	119.1
C16—Ru1—C15	37.1 (2)	C10—C11—H11	119.1
N3—Ru1—C13	153.9 (2)	C17—C12—C13	116.3 (6)
N1—Ru1—C13	129.9 (2)	C17—C12—C19	122.2 (6)
C16—Ru1—C13	78.4 (2)	C13—C12—C19	121.5 (6)
C15—Ru1—C13	66.9 (3)	C17—C12—Ru1	70.6 (3)
N3—Ru1—C17	93.25 (19)	C13—C12—Ru1	70.0 (3)
N1—Ru1—C17	151.50 (19)	C19—C12—Ru1	131.4 (4)
C16—Ru1—C17	37.4 (2)	C14—C13—C12	121.9 (6)
C15—Ru1—C17	67.1 (2)	C14—C13—Ru1	72.7 (4)
C13—Ru1—C17	65.9 (2)	C12—C13—Ru1	72.4 (3)
N3—Ru1—C14	158.2 (2)	C14—C13—H13	118.6
N1—Ru1—C14	103.0 (2)	C12—C13—H13	118.6
C16—Ru1—C14	66.1 (3)	Ru1—C13—H13	118.6
C15—Ru1—C14	37.3 (3)	C13—C14—C15	121.3 (7)
C13—Ru1—C14	36.1 (3)	C13—C14—Ru1	71.2 (4)
C17—Ru1—C14	77.5 (2)	C15—C14—Ru1	70.6 (4)
N3—Ru1—C12	116.54 (19)	C13—C14—H14	118.5
N1—Ru1—C12	167.39 (19)	C15—C14—H14	118.5
C16—Ru1—C12	67.3 (2)	Ru1—C14—H14	118.5
C15—Ru1—C12	80.3 (2)	C16—C15—C14	117.4 (7)
C13—Ru1—C12	37.6 (2)	C16—C15—C18	121.3 (7)
C17—Ru1—C12	36.5 (2)	C14—C15—C18	121.2 (7)
C14—Ru1—C12	66.6 (2)	C16—C15—Ru1	70.5 (3)
N3—Ru1—Cl1	87.39 (12)	C14—C15—Ru1	72.2 (4)
N1—Ru1—Cl1	83.92 (13)	C18—C15—Ru1	127.9 (5)
C16—Ru1—Cl1	159.70 (18)	C15—C16—C17	121.1 (6)
C15—Ru1—Cl1	150.8 (2)	C15—C16—Ru1	72.4 (4)
C13—Ru1—Cl1	91.00 (19)	C17—C16—Ru1	72.5 (3)
C17—Ru1—Cl1	122.36 (16)	C15—C16—H16	119.1
C14—Ru1—Cl1	114.3 (2)	C17—C16—H16	119.1
C12—Ru1—Cl1	93.73 (15)	Ru1—C16—H16	119.1
N2—N1—C1	112.6 (4)	C12—C17—C16	121.9 (6)
N2—N1—Ru1	118.0 (3)	C12—C17—Ru1	72.8 (3)
C1—N1—Ru1	129.4 (3)	C16—C17—Ru1	70.1 (3)
N1—N2—C7	114.5 (4)	C12—C17—H17	118.3
C11—N3—C7	119.4 (4)	C16—C17—H17	118.3
C11—N3—Ru1	128.0 (4)	Ru1—C17—H17	118.3
C7—N3—Ru1	112.7 (3)	C15—C18—H18A	109.5
C8—N4—H4A	120.0	C15—C18—H18B	109.5
C8—N4—H4B	120.0	H18A—C18—H18B	109.5
H4A—N4—H4B	120.0	C15—C18—H18C	109.5
C6—C1—C2	120.0 (5)	H18A—C18—H18C	109.5
C6—C1—N1	120.9 (5)	H18B—C18—H18C	109.5
C2—C1—N1	119.0 (5)	C12—C19—C21	108.7 (9)
C3—C2—C1	119.8 (6)	C12—C19—C20	115.0 (7)
C3—C2—H2	120.1	C21—C19—C20	111.3 (10)
C1—C2—H2	120.1	C12—C19—H19	107.2
C4—C3—C2	120.2 (6)	C21—C19—H19	107.2
C4—C3—H3	119.9	C20—C19—H19	107.2
C2—C3—H3	119.9	C19—C20—H20A	109.5
C3—C4—C5	119.8 (6)	C19—C20—H20B	109.5
C3—C4—H4	120.1	H20A—C20—H20B	109.5
C5—C4—H4	120.1	C19—C20—H20C	109.5
C6—C5—C4	120.0 (7)	H20A—C20—H20C	109.5
C6—C5—H5	120.0	H20B—C20—H20C	109.5
C4—C5—H5	120.0	C19—C21—H21A	109.5
C5—C6—C1	120.0 (6)	C19—C21—H21B	109.5
C5—C6—H6	120.0	H21A—C21—H21B	109.5
C1—C6—H6	120.0	C19—C21—H21C	109.5
N3—C7—N2	119.0 (4)	H21A—C21—H21C	109.5
N3—C7—C8	122.7 (5)	H21B—C21—H21C	109.5
N2—C7—C8	118.2 (4)	H20D—C20B—H20E	109.5
N4—C8—C9	121.4 (5)	H20D—C20B—H20F	109.5
N4—C8—C7	123.0 (5)	H20E—C20B—H20F	109.5
C9—C8—C7	115.7 (5)	H21D—C21B—H21E	109.5
C10—C9—C8	119.8 (5)	H21D—C21B—H21F	109.5
C10—C9—H9	120.1	H21E—C21B—H21F	109.5
C8—C9—H9	120.1		
			
C1—N1—N2—C7	176.3 (4)	C19—C12—C13—C14	177.3 (6)
Ru1—N1—N2—C7	−3.0 (6)	Ru1—C12—C13—C14	−55.7 (5)
N2—N1—C1—C6	−19.8 (7)	C17—C12—C13—Ru1	55.0 (4)
Ru1—N1—C1—C6	159.5 (4)	C19—C12—C13—Ru1	−127.0 (5)
N2—N1—C1—C2	162.6 (5)	C12—C13—C14—C15	3.7 (9)
Ru1—N1—C1—C2	−18.2 (7)	Ru1—C13—C14—C15	−51.8 (5)
C6—C1—C2—C3	−5.1 (9)	C12—C13—C14—Ru1	55.5 (5)
N1—C1—C2—C3	172.7 (5)	C13—C14—C15—C16	−3.7 (9)
C1—C2—C3—C4	4.1 (10)	Ru1—C14—C15—C16	−55.8 (5)
C2—C3—C4—C5	−0.3 (10)	C13—C14—C15—C18	176.1 (6)
C3—C4—C5—C6	−2.5 (11)	Ru1—C14—C15—C18	124.0 (6)
C4—C5—C6—C1	1.5 (10)	C13—C14—C15—Ru1	52.1 (5)
C2—C1—C6—C5	2.3 (9)	C14—C15—C16—C17	0.7 (9)
N1—C1—C6—C5	−175.4 (6)	C18—C15—C16—C17	−179.1 (6)
C11—N3—C7—N2	−177.9 (5)	Ru1—C15—C16—C17	−55.9 (5)
Ru1—N3—C7—N2	1.0 (6)	C14—C15—C16—Ru1	56.6 (5)
C11—N3—C7—C8	−0.1 (8)	C18—C15—C16—Ru1	−123.2 (6)
Ru1—N3—C7—C8	178.8 (4)	C13—C12—C17—C16	−2.3 (8)
N1—N2—C7—N3	1.3 (7)	C19—C12—C17—C16	179.7 (5)
N1—N2—C7—C8	−176.6 (4)	Ru1—C12—C17—C16	52.4 (5)
N3—C7—C8—N4	178.4 (5)	C13—C12—C17—Ru1	−54.7 (4)
N2—C7—C8—N4	−3.8 (8)	C19—C12—C17—Ru1	127.4 (5)
N3—C7—C8—C9	−1.8 (7)	C15—C16—C17—C12	2.3 (9)
N2—C7—C8—C9	176.0 (5)	Ru1—C16—C17—C12	−53.6 (5)
N4—C8—C9—C10	−179.2 (6)	C15—C16—C17—Ru1	55.9 (5)
C7—C8—C9—C10	0.9 (8)	C17—C12—C19—C21	81.0 (17)
C8—C9—C10—C11	1.6 (10)	C13—C12—C19—C21	−96.8 (16)
C7—N3—C11—C10	2.9 (9)	Ru1—C12—C19—C21	172.9 (15)
Ru1—N3—C11—C10	−175.8 (5)	C17—C12—C19—C20	−44.5 (14)
C9—C10—C11—N3	−3.7 (10)	C13—C12—C19—C20	137.7 (12)
C17—C12—C13—C14	−0.7 (8)	Ru1—C12—C19—C20	47.4 (13)

### Hydrogen-bond geometry (Å, º)

**Table:**

*D*—H···*A*	*D*—H	H···*A*	*D*···*A*	*D*—H···*A*
N4—H4*A*···Cl2	0.86	2.34	3.200 (5)	176
N4—H4*B*···Cl2^i^	0.86	2.37	3.152 (5)	151

Symmetry code: (i) *x*−1/2, *y*, −*z*+1/2.
