# Supplementary figures and images for: Crystal structure of [3-amino-2-(phenyl­diazenyl)­pyridine]chlorido­(η6-p-cymene)­ruthenium(II) chloride
Source: Acta Crystallogr E Crystallogr Commun. 2015 Sep 26;71(Pt 10):m185–6. doi: 10.1107/S2056989015017466 (PMC4647410; doi:10.1107/S2056989015017466)

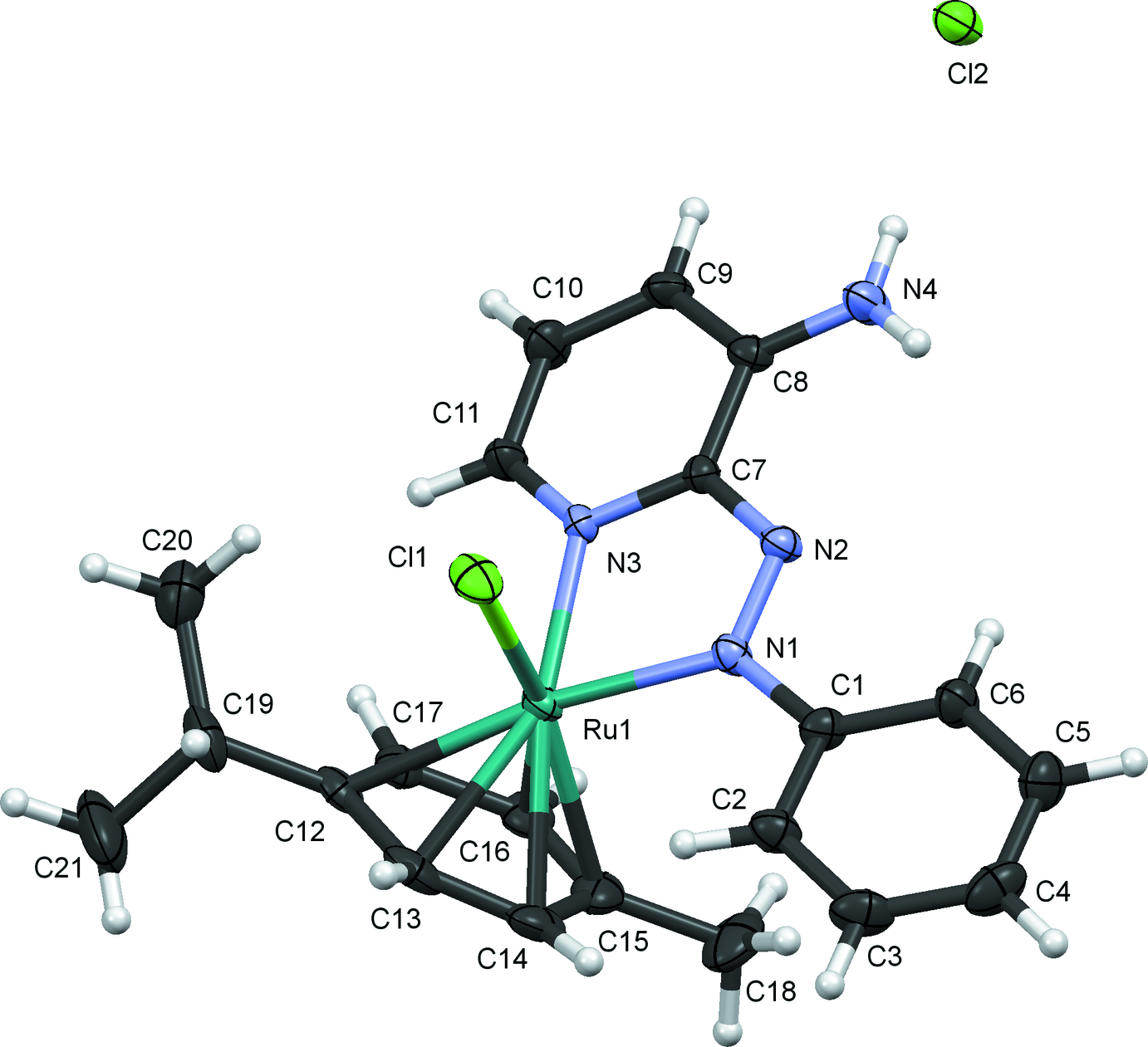

Supplement: Supplementary file 3 [file e-71-0m185-fig1.tif]

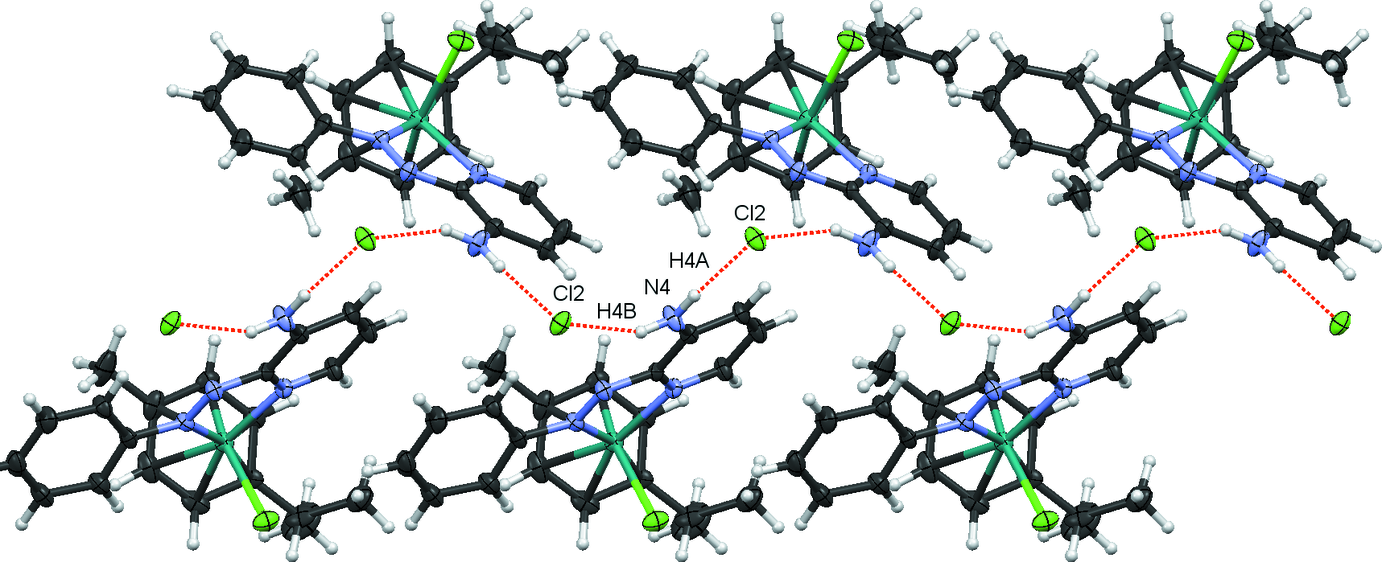

Supplement: Supplementary file 4 [file e-71-0m185-fig2.tif]
